# Supplementary material for: Identification of Freezing-Responsive microRNAs and Their Targets in Chinese Jujube by Small RNA and Degradome Sequencing
Source: Int J Mol Sci. 2026 Sep 4;27(17):7912. doi: 10.3390/ijms27177912 (PMC13566890; doi:10.3390/ijms27177912)
Supplement: Supplementary file 1 [file ijms-27-07912-s001.zip › Supplementary Material S2/novel_miR_109.pdf]

Provisional ID	:	novel_miR_109
Score total	:	0.5
Score for star read(s)	:	-1.3
Score for read counts	:	0
Score for mfe	:	0.2
Score for randfold	:	1.6
Score for cons. seed	:	
Total read count	:	2467
Mature read count	:	1602
Loop read count	:	0
Star read count	:	865

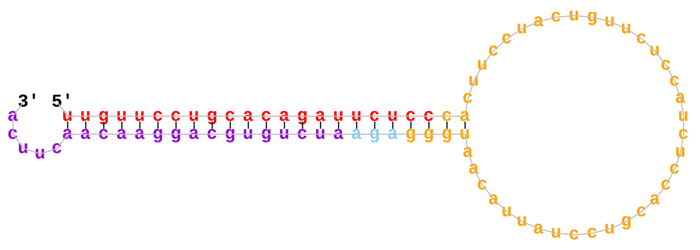

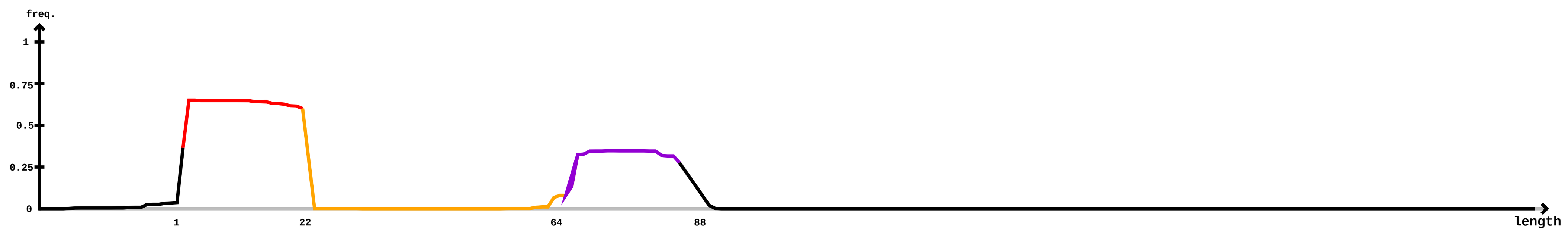

Mature **Star**

```
5'- gaucuuguugauauuguugaaguuguuccugcacagauucucccacuuccuacuguucuccaucuccacguccuauuacaaugggagaaucugugcaggaacaacuucaacaannnnnnnnnnnnnnnnnn...  -3'  obs
    gaucuuguugauauuguugaaguuguuccugcacagauucucccacuuccuacuguucuccaucuccacguccuauuacaauggggagaaucugugcaggaacaacuucaacaannnnnnnnnnnnnnnnnn...       exp
    .(((.....))).((((((((((((((((((((((((((((((.................................))))))))))))))))))))))))))))))................   reads   mm   sample
    ...cuuguugauauuguugaagAug.........................   1    1    JCK-1
    ...cuuguugauauuguugaagAug.........................   4    1    JCK-2
    ....uuguugauauuguugaag............................   3    0    LT-1
    ....uuguugauauuguugaag............................   2    0    LT-3
    .....uguugauauuguugaagAug.........................   1    1    LT-3
    ...........uNuuguugaaguuguuccugc..................   1    1    JCK-2
    .............uuguugaaguuguuccugca.................   1    0    LT-3
    .............uuguugaaguuguuccugcac................   2    0    LT-1
    .............uuguugaaguuguuccugcac................   1    0    LT-3
    .............uuguugaaguuguuccugcac................   4    0    JCK-3
    ..............uguugaaguuguuccugcaca...............   1    0    LT-1
    ................uugaaguuguuccugcac................   3    0    JCK-1
    ................uugaaguuguuccugcac................   1    0    LT-3
    ................uugaaguuguuccugcac................   3    0    LT-1
    ................uugaaguuguuccugcac................   1    0    JCK-3
    ................uugaaguuguuccugcacag..............   1    0    JCK-3
    ................uugaaguuguuccugcacag..............   1    0    JCK-2
    ................uugaaguuguuccugcacag..............   1    0    LT-3
    ................uugaaguuguuccugcacaga.............   5    0    JCK-3
    ................uugaaguuguuccugcacaga.............   2    0    LT-2
    ................uugaaguuguuccugcacaga.............   10   0    JCK-2
    ................uugaaguuguuccugcacaga.............   5    0    JCK-1
    ................uugaaguuguuccugcacaga.............   1    0    LT-3
    ................uugaaguuguuccugcacagauu...........   7    0    JT-3
    ................uugaaguuguuccugcacagauC...........   1    1    LT-1
    ................uugaaguuguuccugcacagauu...........   3    0    JT-2
    .................ugaaguuguuccugcacaga.............   1    0    JCK-3
    .................ugaaguuguuccugcacagau............   1    0    JCK-3
    ...................aaguuguuccugcacagauuc..........   5    0    JCK-2
    ...................aaguuguuccugcacagauuc..........   8    0    JCK-3
    ...................aaguuguuccugcacagauuc..........   3    0    JCK-1
    ....................aguuguuccugcacagauu...........   1    0    LT-2
    ....................aguuguuccugcacagauuc..........   2    0    JCK-3
```

Mature **Star**

```
gaucuuguugauauuguugaaguuguuccugcacagauucucccacuuccuacuguucuccaucuccacguccuauuacaaugggagaaucugugcaggaacaacuucaacaannnnnnnn…
....................aguuguuccugcacagauuc.…   1    0    JCK-2
....................aguuguuccugcacagauucu.…   1    0    JCK-2
.....................Cuuguuccugcacagauucucc.…   1    1    JCK-1
.....................Cuuguuccugcacagauucucc.…   1    1    JCK-3
.....................Cuuguuccugcacagauucucc.…   3    1    JCK-2
......................uuguuccugcacagauuc.…   2    0    JCK-1
......................uuguuccugcacagauuU.…   2    1    JCK-1
......................uuguuccugcacagauuc.…   1    0    JCK-3
......................uuguuccugcacagauuU.…   1    1    JCK-3
......................uuguuccugcacagauucu.…   1    0    JCK-1
......................uuguuccugcacagauucu.…   1    0    JCK-2
......................uuguuccugcacagauucuc.…   4    0    LT-2
......................uuguuccugcacagauucuc.…   2    0    JCK-3
......................uuguuccugcacagauucuc.…   1    0    JCK-1
......................uuguuccugcacagauucuc.…   3    0    JCK-2
......................uuguuccugcacagCuucucc.…   1    1    JCK-3
......................uuguuccugcacagauucucc.…   291  0    JCK-2
......................uuguuccugcacagauucucc.…   26   0    LCK-3
......................uuguuccugcacagauucucc.…   13   0    JT-1
......................uuguuccugcacCgauucucc.…   1    1    JCK-1
......................uuguuccugcaNagauucucc.…   1    1    JCK-1
......................uuUuuccugcacagauucucc.…   1    1    JCK-2
......................uuguuccugcacagauucucc.…   28   0    LT-1
......................uuguuccugcCcagauucucc.…   1    1    JCK-2
......................uuguuccugcacagauucucc.…   215  0    JCK-3
......................uuguuccugcacCgauucucc.…   1    1    JCK-3
......................uuguuccugcacagauucucc.…   28   0    LT-2
......................uuguuccugcacagauucucc.…   52   0    LT-3
......................uNguuccugcacagauucucc.…   1    1    JCK-3
......................uuguucAugcacagauucucc.…   1    1    JCK-3
......................uuguuccugcacagauucuUc.…   1    1    JCK-2
......................uugNuccugcacagauucucc.…   1    1    JCK-1
......................uuguuccugcacagauucucc.…   174  0    JCK-1
.......................uguuccugcacagauucu.…   1    0    LT-3
.......................uguuccugcacagauucuc.…   2    0    LT-1
.......................uguuccugcacagauucuc.…   4    0    JCK-1
.......................uguuccugcacagauucuc.…   4    0    LCK-3
.......................uguuccugcacagauucuc.…   3    0    LT-2
.......................uguuccugcacagauucuc.…   4    0    JCK-2
.......................uguuccugcacagauucuc.…   3    0    LT-3
.......................uguuccugcacagauucuc.…   2    0    JT-2
.......................uguuccugcacagauucuc.…   3    0    JCK-3
.......................uguuccugcacagauucuc.…   2    0    JT-3
.......................uguuccugcacagauucucc.…   12   0    JCK-2
.......................uguuccugcacagauucucU.…   1    1    JCK-2
.......................uguuccugcacagauucucc.…   4    0    LT-3
.......................uguuccugcacagauucucU.…   1    1    LT-2
.......................uguuccugcacagauucucU.…   1    1    JCK-3
.......................uguuccugcacagauucucc.…   13   0    LCK-3
.......................uguuccugcacagauucucU.…   1    1    LT-3
.......................uguuccugcacagauucucc.…   1    0    LT-2
.......................uNuuccugcacagauucucc.…   1    1    JCK-3
.......................uguuccugcacagauucucc.…   5    0    JCK-1
.......................uguuccugcacagauucucc.…   5    0    JCK-3
.......................uguuccugcacagauucuccc.…   11   0    LCK-2
.......................uguuccugcacagauucuccc.…   171  0    JCK-3
.......................uguuccugcacagauucuccc.…   24   0    LT-3
.......................uguuccuAcacagauucuccc.…   1    1    JCK-3
.......................uguuccugcCcagauucuccc.…   1    1    JCK-1
.......................uguuccugcacagauucuccc.…   13   0    LT-2
.......................uguuccugcacagauucuccc.…   13   0    LCK-3
.......................uguuccugcacagauucuccc.…   133  0    JCK-1
.......................uguuccugcacagauucuccc.…   5    0    JT-2
.......................uguuccugcacagauucuccc.…   236  0    JCK-2
.......................uguuccugcacagauucuccc.…   12   0    LCK-1
.......................uguuccugcacaAauucuccc.…   1    1    JCK-2
.......................uguuccugcacagauucuccc.…   11   0    JT-3
.......................ugCuccugcacagauucuccc.…   1    1    JCK-3
.......................uNuuccugcacagauucuccc.…   1    1    JCK-3
.......................uguuccugcacagauucuccG.…   1    1    JCK-3
```

Mature Star

gaucuuguugauauuguugaaguuguuccugcacagauucucccacuuccuacuguucuccaucuccacguccuauuacaaugggagaaucugugcaggaacaacuucaacaannnnnnnnnnnnnnnnnnnnnnnnnnnnnnnnnnnnnnnnnnnnnnnnnnnnnnnnnnnnnnnnnnnnnnnnnnnnnnnnnnnnnnnnnnnnnnnnnnnnnnnnnnnnnnnnn

uguuccugcacagauucuccc	24	0	LT-1
uguuccugAacagauucuccc	5	1	LT-3
uguuccugcacagauucucccacuuccua	2	0	JT-2
uacaaugggagaaucugugcagga	2	0	LT-2
acaaugggagaaucugug	1	0	LT-3
ugggagaaucugugcaggaac	3	0	JCK-2
ugggagaaucugugcaggaac	7	0	LT-2
ugggagaaucugugcaggaac	2	0	JCK-3
ugggagaaucugugcaggaac	1	0	LT-3
ugggagaaucugugcaggaacaac	2	0	JT-2
ugggagaaucugugcaggaacaac	3	0	JT-3
gggagaaucugugcaggaacaac	6	0	JT-1
gggagaCucugugcaggaacaac	1	1	JT-1
gagaaucugugcaggaac	10	0	JCK-2
gagaaucugugcaggaac	17	0	LCK-1
gagaaucugugcaggaac	11	0	LCK-2
gagaaucugugcaggaac	3	0	JCK-1
gagaaucugugcaggaac	6	0	LT-3
gagaaucugugcaggaac	3	0	LT-2
gagaaucugugcaggaac	4	0	LT-1
gagaaucugugcaggaaca	7	0	JT-1
gagaaucugugcaggaaca	1	0	JCK-2
gagaaucugugcaggaaca	1	0	JCK-3
gagaaucugugcaggaacaac	3	0	LT-1
gagaaucugugcaggaacaac	19	0	JCK-3
gagaaucugugcaggaacaac	2	0	LT-2
gagaaucugugcaggaacaac	11	0	LT-3
gagaaucugugcaggaacaac	7	0	LCK-3
gagaaucugugcaggaacaac	29	0	JCK-2
gagaaucugugcaggaacaac	10	0	JCK-1
agaaucugugcaggaacaac	2	0	JCK-2
agaaucugugcaggaacaac	1	0	LT-3
agaaucugugcaggaacaac	2	0	JCK-1
agaaucugugcaggaacaac	1	0	LT-1
agaaucugugcaggaacaacu	4	0	LT-1
agaaucugugcaggaacaacu	5	0	JT-2
agaaucugugcaggaacaacu	2	0	JCK-2
agaaucugugcaggaacaacu	3	0	LT-3
agaaucugugcaggaacaacu	4	0	LT-2
agaaucugugcaggaacaacu	1	0	JCK-1
agaaucugugcaggaacaacu	3	0	JT-3
agaaucugugcaggaacaacu	2	0	JCK-3
agaaucugugcaggaacaacu	5	0	LCK-3
aaucugugcaggaaUaac	6	1	LCK-3
aaucugugcaggaacaacu	1	0	LCK-3
aaucugugcaggaacaacu	1	0	LT-1
aaucugugcaggaacaacu	1	0	JCK-2
aaucugugcaggaacaacuu	2	0	LT-1
aaucugugcaggaacaacuu	2	0	LT-3
aaucugugcaggaacaacuuc	11	0	LT-3
aaucugugcaggaacaacuuc	4	0	LT-1
aaucugugcaggaacaacuuc	39	0	JCK-2
aaucugugcaggaacaacuuc	4	0	LCK-3
aaucugugcCggaacaacuuc	1	1	JCK-2
aaucugugcaggaacaacuuc	29	0	JCK-3
aaucugugcaggaacaacuuc	26	0	JCK-1
aaucugugcaggaacaacuuca	4	0	LCK-3
aaucugugcaggaacaacuuca	3	0	JCK-2
aaucugugcaggaacaacuuAa	2	1	JCK-3
aaucugugcaggaacaacuuca	3	0	LT-3
aaucugugcaggaacaacuuca	2	0	JCK-3
aaucugugcaggaacaacuuca	1	0	LT-1
aucugugcaggaacaacu	2	0	LT-2
aucugugcaggaacaacu	1	0	LT-3
aucugugcaggaacaacu	1	0	JCK-2
aucugugcaggaacaacu	4	0	LCK-3
aucugugcaggaacaacuu	20	0	JT-1
aucugugcaggaacaacuu	1	0	LT-3
aucugugcaggaacaacuuc	21	0	JCK-3
aucugugcaggaacaacuuc	16	0	LT-3

Mature **Star**

gaucuuguugauauuguugaaguuguuccugcacagauucucccacuuccuacuguucuccaucuccacguccuauuacaaugggagaaucugugcaggaacaacuucaacaannnnnnnnnnnnnnnnnnnnnnnnnnnnnnnnnnnnnnnnnnnnnnnnnnnnnnnnnnnnnnnnnnnnnnnnnnnnnnnnnnnnnnnnnnnnnnnnnnnnnnnnnnnnnnnnnnnnnnnnnnnnnnnn

aucugugcaggaacaacuuc	20	0	JCK-2
aucugugcaggaacaacuuc	15	0	LT-1
aucugugcaggaacaacuuc	20	0	JCK-1
aucugugcaggaacaacuuc	22	0	LT-2
aucugugcaggaacaacuuca	28	0	LCK-3
aucugugcaggaacaacuuca	120	0	JCK-2
aucugugcaggaacaacuuca	12	0	LT-1
aucugugcaggGacaacuuca	1	1	JCK-2
aucugugcaggaacaacuuca	70	0	JCK-1
aNcugugcaggaacaacuuca	1	1	JCK-3
aucugugcaggaacaacuuca	73	0	JCK-3
aucugugUaggaacaacuuca	1	1	JCK-2
aucugugcaggaacaacuuca	20	0	LT-3
aucugugcaggaacaacuuca	19	0	LT-2
aucugugcaggaacaacuucaac	1	0	JCK-2
aucugugcaggaacaacuucaac	1	0	JCK-1
ucugugcaggaacaacuuc	2	0	LCK-3
ucugugcaggaacaacuuc	1	0	JCK-3
ucugugcaggaacaacuuca	1	0	JCK-1
ucugugcaggaacaacuuca	2	0	JCK-3
ucugugcaggaacaacuucaac	1	0	JCK-2
cugugcaggaacaacuuc	3	0	JCK-3
cugugcaggaacaacuuca	1	0	JCK-2
cugugcaggaacaacuucaac	3	0	LT-1
cugugcaggaacaacuucaac	10	0	JCK-1
cugugcaggaacaacuucaac	13	0	JCK-2
cugugcaggaacaacuucaac	12	0	JCK-3
cugugcaggaacaacuucaac	1	0	LT-3
cugugcaggaacaacuucaacU	1	1	LT-3
cugugcaggaacaacuucaacU	2	1	JCK-3
cugugcaggaacaacuucaacU	1	1	JCK-2
ugugcaggaacaacuucaac	1	0	JCK-2
ugcaggaacaacuucaac	3	0	LT-1
